# Interference with the Cannabinoid Receptor CB1R Results in Miswiring of GnRH3 and AgRP1 Axons in Zebrafish Embryos

**DOI:** 10.3390/ijms21010168

**Published:** 2019-12-25

**Authors:** Giulia Zuccarini, Ilaria D’Atri, Erika Cottone, Ken Mackie, Inbal Shainer, Yoav Gothilf, Paolo Provero, Patrizia Bovolin, Giorgio Roberto Merlo

**Affiliations:** 1 Department of Life Sciences and Systems Biology, University of Torino, 10123 Torino, Italy; giulia.zuccarini@edu.unito.it (G.Z.); id246@exeter.ac.uk (I.D.); erika.cottone@unito.it (E.C.); 2 Department of Molecular Biotechnologies and Health Sciences, University of Torino, 10126 Torino, Italy; paolo.provero@unito.it; 3 Wellcome Wolfson Centre for Medical Research, University of Exeter, Exeter EX2 5DW, UK; 4 Department of Psychological and Brain Sciences, Gill Center for Biomolecular Science, Indiana University, Bloomington, IN 47405, USA; kmackie@indiana.edu; 5 Department of Neurobiology, The George S. Wise Faculty of Life Sciences and Sagol School of Neuroscience, Tel-Aviv University, Tel Aviv 6997801, Israel; shainer@neuro.mpg.de (I.S.); yoavgothilf@gmail.com (Y.G.)

**Keywords:** cannabinoid receptor, GnRH, AgRP, neuroendocrine, axon guidance, stathmin, zebrafish

## Abstract

The G protein-coupled cannabinoid receptors type 1 (CB1R) and type 2 (CB2R), and their endocannabinoid (eCBs) ligands, have been implicated in several aspects of brain wiring during development. Here we aim to assess whether interfering with CB1R affects development, neuritogenesis and pathfinding of GnRH and AgRP neurons, forebrain neurons that control respectively reproduction and appetite. We pharmacologically and genetically interfered with CB1R in zebrafish strains with fluorescently labeled GnRH3 and the AgRP1 neurons. By applying CB1R antagonists we observed a reduced number of GnRH3 neurons, fiber misrouting and altered fasciculation. Similar phenotypes were observed by CB1R knockdown. Interfering with CB1R also resulted in a reduced number, misrouting and poor fasciculation of the AgRP1 neuron’s axonal projections. Using a bioinformatic approach followed by qPCR validation, we have attempted to link CB1R functions with known guidance and fasciculation proteins. The search identified stathmin-2, a protein controlling microtubule dynamics, previously demonstrated to be coexpressed with CB1R and now shown to be downregulated upon interference with CB1R in zebrafish. Together, these results raise the likely possibility that embryonic exposure to low doses of CB1R-interfering compounds could impact on the development of the neuroendocrine systems controlling sexual maturation, reproduction and food intake.

## 1. Introduction

Marijuana and other derivatives of the plant *Cannabis* spp. have been used for thousands of years for their therapeutic and mood-altering/recreational properties. *Cannabis*-derived psychoactive compounds, mainly ∆9-tetrahydrocannabinol (∆9-THC) and cannabidiol (CBD), can pass the placental barrier and be transferred to the embryo [1]. It is now recognized that *Cannabis* consumption during pregnancy can exert adverse consequences on the progeny, including anxiety, cognitive and attention deficits, as well as depression [2].

In the central nervous system (CNS), ∆9-THC, CBD and various other synthetic cannabinoids bind the G protein-coupled receptors of the cannabinoid family, CB1R and CB2R [3]. The cognate endogenous ligands, known as endocannabinoids (eCBs), are small signaling lipids synthesized “on demand” from membrane lipids. The most abundant eCB in the CNS is 2-arachidonoylglycerol (2-AG), derived from the cleavage of diacylglycerol by diacyl-glycerol lipase (DAGL) and degraded by the action of monoacylglycerol lipase (MAGL) [4,5,6]. eCBs are mainly recognized by the CB1 and CB2 receptors, although they can also bind to other receptor types (TRP, PPARs, etc.). During CNS development, CB1R expression levels gradually increase and are localized on developing axonal projections, while CB2Rs are rather expressed by uncommitted precursor cells [7]. The localization of the eCB-synthesizing enzyme DAGL usually overlaps with that of CB1Rs in preterminal axons and growth cones, while MAGL is actively excluded from growth cones until synaptogenesis is concluded [8]. Upon release, eCBs have been shown to act in both autocrine and paracrine fashion and can cross cell membranes. In contrast to the primarily autocrine mechanism of action in the immature CNS, in the adult synapses it has been shown that eCBs act as retrograde signals (released post-synaptically, and acts on pre-synaptic termini to modulate neurotransmitter release) through a paracrine mechanism.

Following binding of eCBs, phyto-CBs and synthetic CBs to CB1R, a variety of transduction pathways are activated in a time- and region-dependent way, depending on the cell type and receptor repertoires. In particular, eCBs have been proposed to activate Rho-class of guanosine tri-phosphate phosphatases (Rho-GTPases) and to control the neuronal actomyosin cytoskeleton [9]. It has been demonstrated that [10] direct interaction of CB1 with the WAVE1/RAC1 complex, which acts on actin-binding proteins, results in actin remodeling and growth cone dynamics. Activated CB1R can also modulate microtubule dynamics at the growth cone, via the c-Jun NH(2)-terminal kinases (JNKs) pathway [11,12,13]. By modulating the actomyosin network and microtubule dynamics at the growth cone [9], in addition to axon growth and guidance, CB1 receptors have been shown to participate in the control of synaptogenesis and synaptic plasticity in various experimental models [14,15,16].

Data from the medical literature support the role of CB1R in brain wiring during early development. Fetuses exposed to cannabis during embryonic life have increased risk of cognitive deficit [17,18], attention deficit [19] and anxiety and depression [20], as well as defects in neuronal migration and axonal pathfinding in several brain districts [2,21]. Most experimental studies have focused mainly on the cerebral cortex, the hippocampus and the visual system in mice, where CB1R regulates axon guidance and map formation [22,23,24,25,26,27,28]. Importantly, exposure of pregnant mice to ∆9-THC results in life-long circuit modification and altered wiring in the offspring [29]. In zebrafish embryos, interfering with CB1R signaling has been shown to result in abnormal axonal growth and fasciculation in the anterior and posterior commissures of the forebrain. Moreover, exuberant axons of reticulospinal neurons in the hindbrain were found to cross the midline or to deviate from their trajectory and turn backward [30]. Similarly, the selective pharmacological inhibition of CB1R and CB2R in early embryos resulted in altered development of the locomotor system at later stages [31], and treatment with ∆9-THC or CBD resulted in altered motor neurons morphology, synaptic activity at the neuromuscular junction and locomotor responses to sound [32]. Reducing the level of 2-AG via knockdown of *Daglα* resulted in abnormal behaviors, characterized by stereotyped movement and altered motion perception [33].

Few reports have linked the eCB/CB1R signaling functions to the maturation and physiology of the hypothalamic neuroendocrine systems controlling reproduction and appetite [34,35,36]. The gonadotropin releasing hormone (GnRH) neurons, which are central for the control of sexual maturation, fertility and reproduction, have been extensively studied. In this context, eCBs have been shown to modulate the input and the firing activity of GnRH neurons [36], and CB1R activation inhibits presynaptic GABA release [37,38]. Accordingly, the literature reports a link between perturbations of CB1R (either due to consumption of phyto-cannabinoids or exposure to synthetic cannabinoids) and altered functionality of the neuroendocrine gonadotropic system, puberty and fertility deficits [35,36].

Another important prominent effect of the eCB system is the regulation of appetite and energy balance. The hypothalamic network of peptidergic neurons controlling food intake/energy metabolism, enhancing feeding behavior, includes mainly neurons expressing pro-opiomelanocortin (POMC), neuropeptide Y (NPY) and agouti-related peptide (AgRP) [39]. This system is highly conserved among vertebrates. Pharmacological blockade of CB1R suppresses hunger and induces hypophagia, and the CB1R-antagonist Rimonabant in the past has been proposed for the treatment of obesity [40,41,42]. Importantly, eCB/CB1R signaling has been shown to have an impact on AgRP/NPY neurons [43] and indeed its perturbation has been associated with altered appetite control, energy storage/consumption and metabolic conditions [44].

While most previous works have focused on postnatal and adult hypothalamic functions, little is known on the role of CB1R and its misactivation for the correct development of hypothalamic neuroendocrine neurons and their connectivity needed to attain control of sexual maturation and/or reproduction and appetite/energy storage. This question is highly relevant, to fully assess the impact of exposure to these (and other molecules) during embryonic and early infancy on human health, and the possible link with adult pathologies, such as reduced fertility and altered food intake. Furthermore, these studies aimed to elucidate the role of eCB ligands and the mechanisms underlying axonal miswiring upon altered CB1R activation [7].

We have investigated the possible role of CB1R altered activity on the extension, fasciculation and pathfinding of GnRH3 and AgRP axons during forebrain development in zebrafish. Using transgenic lines in which GnRH3 and AgRP neurons are fluorescently labeled, we show that pharmacological and genetic manipulation of CB1R resulted in abnormal guidance, fasciculation and routing of GnRH3 and AgRP1 axons during embryonic development, indicating that CB1R is a possible regulator of the development of reproduction- and food intake-related neurons.

## 2. Results

### 2.1. Survival and Hatching Rates of Zebrafish Embryos Treated with CB1R Ligands

In order to select a range of non-toxic concentrations and non-teratogenic doses of CB1R agonists and antagonists, we initially determined their effects on the survival and hatching rates of zebrafish embryos. *gnrh3::EGFP* [45,46] and *agrp1::mCherry* [47] zebrafish embryos were exposed to different compounds starting at 0 hours post fertilization (hpf). Hatching rate was examined at 48 hpf and 72 hpf for *gnrh3::EGFP* embryos and also at 96 hpf for *agrp1::mCherry* embryos. The survival rate was examined at 24 hpf, 48 hpf and 72 hpf for *gnrh3::EGFP* embryos and at 24 hpf, 48 hpf, 72 hpf and 96 hpf for *agrp1::mCherry* embryos. Survival rate of *agrp1::mCherry* embryos at 96 hpf was analyzed as well because at 72 hpf the fluorescence signal of the AgRP1 fibers was still too weak to be reliably evaluated by fluorescence microscopy. The survival rate was calculated as the percentage of live embryos over the total number of embryos (30 embryos/well for each concentration); the hatching rate was calculated as the percentage of hatched embryos over the remaining living embryos.

As agonist of the CB1 receptor, we chose WIN55,212-2 (renamed WIN55 for simplicity), which has been reported to mimic endocannabinoids (eCBs) signaling in several studies [22,48,49,50]. Four concentration of WIN55 were tested: 1 nM, 10 nM, 100 nM and 1 µM [48,50]. As antagonist/inhibitors of CB1R we chose Rimonabant and AM251, known to act on CB1R as inverse agonist or antagonist, respectively [9,22,23,48]. Four concentrations for Rimonabant (1 nM, 10 nM, 100 nM and 1 µM) and three concentrations for AM251 (100 nM, 1 µM and 5 µM) were tested.

**Figure 1:**
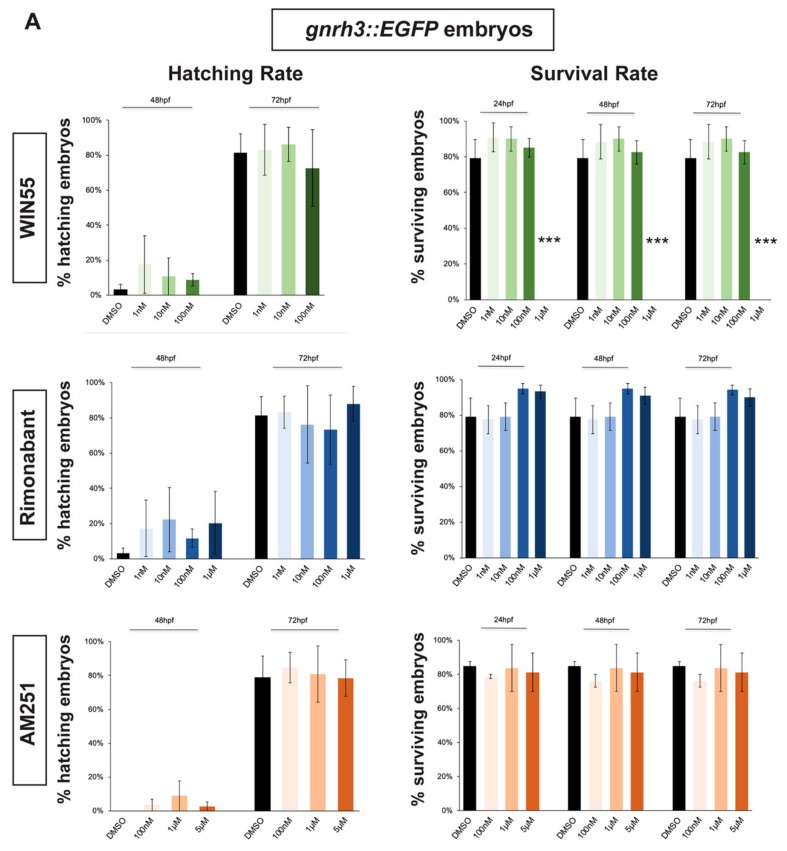

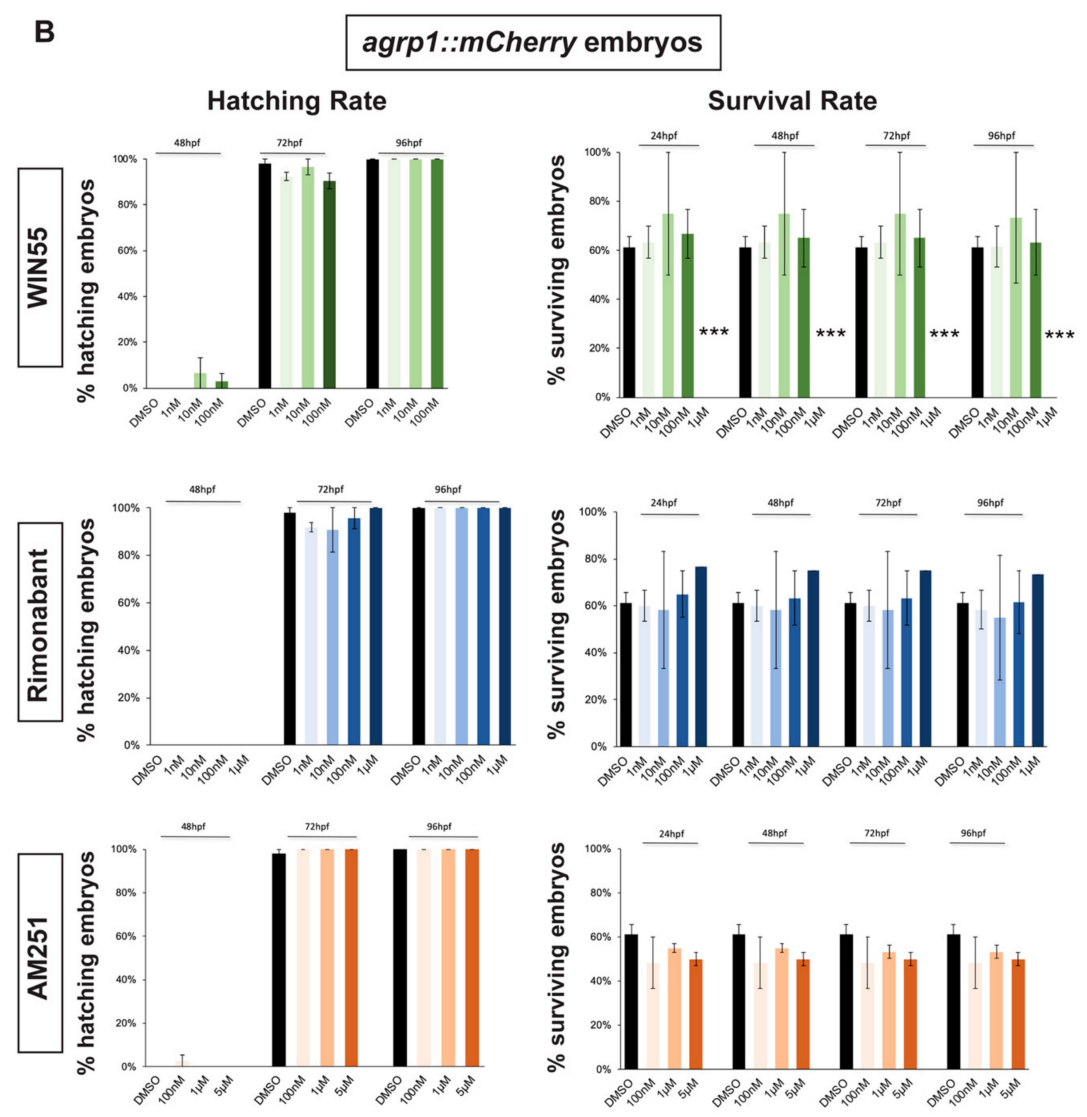
Survival and hatching rates in zebrafish embryos treated with CB1R ligands. (**A**) Hatching rate (panels on the left) and survival rate (panels on the right) of *gnrh3::EGFP* zebrafish embryos following treatments with WIN55, Rimonabant or AM251, at the indicated doses, examined at the indicated embryonic age. Results are expressed as % over the total number of embryos examined. (**B**) Same as in (**A**), but using *agrp1::mCherry* embryos. *** = *p* < 0.001.

Hatching, normally occurring between 48 and 72 hpf, was not affected by the exposure to any concentration of CB1R ligand in both *gnrh3::EGFP* and *agrp1::mCherry* embryos (Figure 1A,B). Concerning the survival rate, we observed that the highest WIN55 concentration (1 µM) caused complete embryonic lethality for both *gnrh3::EGFP* and *agrp1::mCherry* embryos, already at 24 h, while the survival of both transgenic lines following different concentrations of AM251 and Rimonabant was unchanged as compared to the DMSO-treated control embryos (Figure 1A,B).

### 2.2. Effects of CB1R Ligands on Zebrafish GnRH3 Neurons

In fish embryos, GnRH3 neurons are first seen at 24 hpf in the olfactory placode region, associated with the terminal nerve. By 48 hpf, axons extending from GnRH3 neurons present in the nasal region project dorsally and ventrally into the forebrain and enter the anterior commissure (AC) and the postoptic commissure. In addition, two tracts extend along the optic nerve and innervate the retina, a tract extends dorsally towards the region of the pineal gland and a tract extends posteriorly towards the hypothalamus and preoptic area. Between 3 and 15 days post-fertilization (dpf), soma migration takes place in an axonophilic manner along the terminal nerve and the ventral telencephalon towards the ventral hypothalamus [45]. Some GnRH3 neurons are thought to migrate to the hypothalamus, while others remain along the path [46].

**Figure 2:**
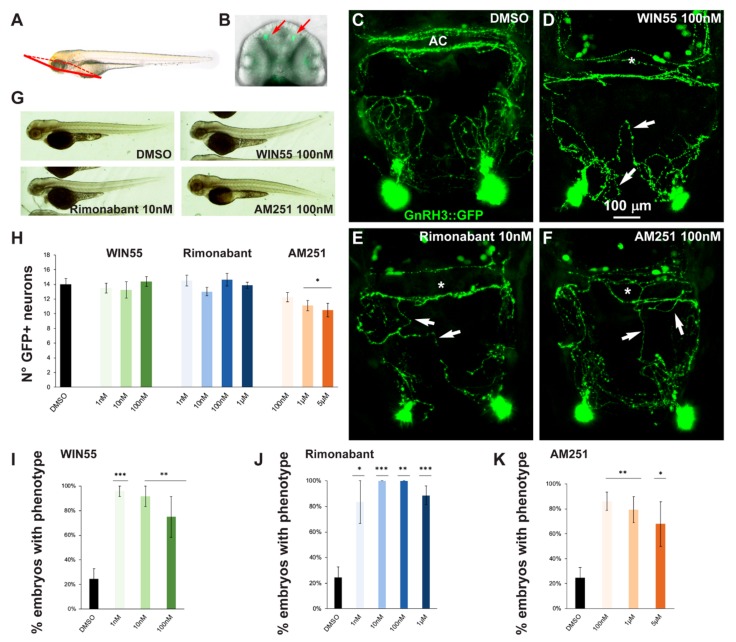
Pharmacological manipulation of CB1R on GnRH3 neurons in zebrafish embryos. (**A**) Scheme showing the observation plane used for the micrographs in (**C–F**). (**B**) Low magnification of the head piece of *gnrh3::EGFP* embryos, viewed in combined fluorescence and bright field illumination. Red arrows indicate the EGFP+ cells associated to the terminal nerve. (**C**–**F**) Representative images of EGFP+ neurons and their projections present in the nasal and basal forebrain regions of embryos at 72 hpf, treated either with DMSO only (negative control; **C**), with WIN55 (**D**), Rimonabant (**E**) or AM251 (**F**), at the indicated doses. Scale bar is reported in **D**. White arrows indicate misguided EGFP+ projections, and white asterisks indicate altered organization of EGFP+ fibers at the anterior commissure. (**G**) Bright-field low magnification images of whole embryos treated with DMSO only, WIN55, Rimonabant or AM251, showing a normal general morphology and growth. (**H**) Quantification of the number of EGFP+ neurons in the nasal region of fish embryos treated with DMSO only (black bar), or with WIN55, Rimonabant or AM251 at the indicated doses. The color code is the same as in Figure 1. No significant difference in the number of EGFP+ neurons was observed following these treatments. (**I–K**) Quantification of the misguidance and altered commissural phenotypes, expressed as % of the number of embryos presenting the phenotype, upon treatment with WIN55 (**I**), Rimonabant (**J**) or AM251 (**K**). Color code as in Figure 1. Data are expressed as means ± SEM from three independent experiments. * = *p* < 0.05; ** = *p* < 0.01; *** = *p* < 0.001. AC, anterior commissure.

In order to assess possible effects of altered CB1R function on the development of the GnRH3 system in the olfactory region and anterior commissure, *gnrh3::EGFP* embryos at the 1-cell stage were treated with various concentrations of CB1R-ligands for 72 h after which the EGFP+ embryos were selected and examined by confocal microscopy. A proper view plane of the transgenic fish was adopted to allow the correct visualization of both GnRH3 neurons present in the olfactory region and GnRH3 fibers extending towards the anterior commissure (shown in Figure 2A,B). As pointed out in the previous section, with all the ligands and all concentrations used in this study we found no evident morphological changes or developmental delay, as shown by low magnification bright field whole embryos examination (Figure 2G).

The number, neurite extension and commissural fasciculation of GnRH3 neurons and fibers were examined and compared to control embryos treated with DMSO only (Figure 2C). We found that ligand exposure led to abnormal axonal pathfinding, with fibers that did not extend with proper directionality (Figure 2D–F, white arrows) and a failure in fasciculation of axonal bundles at the level of the anterior commissure (Figure 2D–F, asterisks). For quantification purposes, we defined as “phenotype” the condition in which we observe a) altered orientation and position of EGFP+ fibers; b) altered fasciculation of EGFP+ fibers at the commissures; or c) both of these together. With this definition, the percentage of embryos showing this phenotype was calculated for each ligand concentration tested (WIN55: 1 nM, *n* = 10 embryos; 10 nM, *n* = 10; 100 nM, *n* = 12; Rimonabant: 1 nM, *n* = 10; 10 nM, *n* = 13; 100 nM, *n* = 10; 1 μM, *n* = 20; AM251: 100 nM, *n* = 12; 1 μM, *n* = 14; 5 μM, *n* = 11) and for the control embryos (DMSO only, *n* = 30). The results were then expressed as the number of embryos with the phenotype in the various conditions, as percentage over the total number of embryos examined, and reported in Figure 2I–K. We observed an increase in the proportion of embryos showing the phenotype after drug treatment (Figure 2I–K). Thus, treatment with either a CB1R-agonist (WIN55) or CB1R-antagonist/inverse agonist (AM251 and Rimonabant) resulted in miswiring of GnRH3 fibers, indicating that either the activation or de-activation of CB1R has similar negative consequences on axonal fasciculation and navigation.

At higher magnification, we also counted the number of EGFP+ cells present in the olfactory region (Figure 2H). We observed no difference between the control embryos and embryos treated with WIN55 and Rimonabant (Figure 2I–J). Conversely, a decrease in cell number was observed with high concentrations of AM251 (Figure 2K), suggesting that the inhibition of CB1R signaling may interfere with GnRH3 cell proliferation or accelerate the migration of GnRH3 neurons to the hypothalamus.

### 2.3. Effects of CB1R Knockdown on Zebrafish GnRH3 Neurons

In order to verify if down-modulation of CB1R could lead to phenotypes similar to the ones observed after treatment with CB1R ligands, we utilized a conventional antisense morpholino oligonucleotide (MO)-mediated strategy. *gnrh3::EGFP* embryos were injected at the 1-cell stage with a previously published *z-cb1r* morpholino, whose sequence was designed to bind to a region 5′ of the ATG start codon and was shown to block *z-cb1r* mRNA translation [30]. Uninjected embryos and embryos injected with a scrambled-sequence MO (Mismatch_PPFIA) were used as controls.

**Figure 3:**
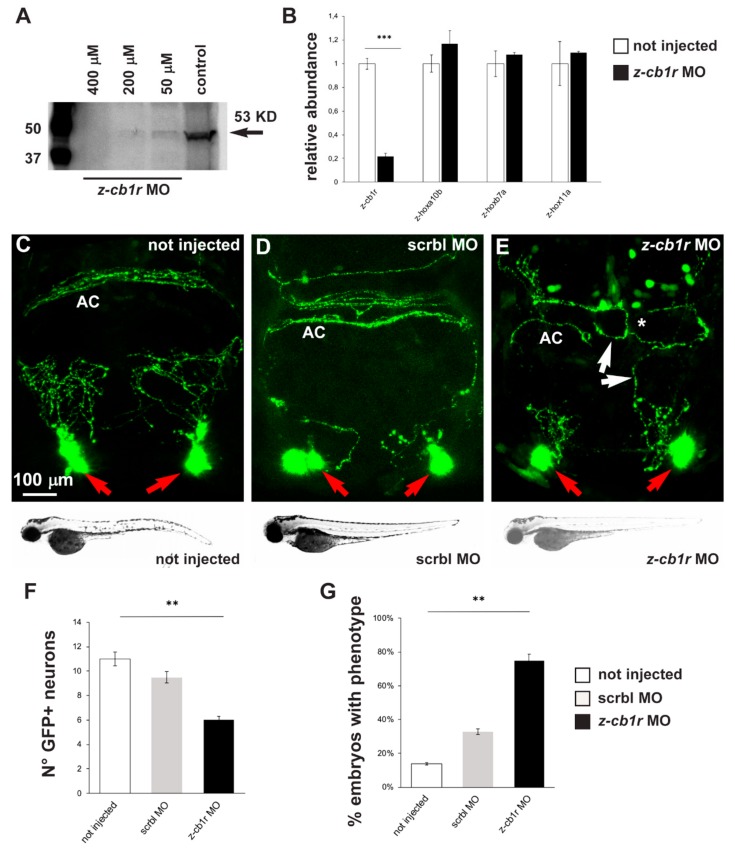
Effects of MO-mediated CB1R knockdown on GnRH3 neurons in zebrafish embryos. (**A**) Western blot analyses of protein extracts from uninjected or *z-cb1r* MO-injected zebrafish embryos. A significant depletion of the CB1R protein was detected in the treated embryos. (**B**) Real-time qPCR analyses of *z-cb1r* and three *z-hox* genes on RNA samples extracted from uninjected (open bars) or *z-cb1r* MO-injected (solid black bars) embryos. Expression is shown relative to the expression of the housekeeping gene mRNA (*z-β-actin*). The abundance of the control RNA is set as 1. A depletion of the *z-cb1r* mRNA in embryos injected with *z-cb1r* MO is observed, as compared to control ones. No change in the expression of *z-hox* genes was observed, indicating unaltered developmental progression. (**C**–**E**) Representative images of EGFP GnRH3 neurons and projections in the nasal and the basal forebrain regions of zebrafish embryos, at the age 72 hpf. The observation plane is the same as in Figure 2. Scale bar is reported in **C**. Red arrows indicate EGFP+ neurons, white arrows indicate altered EGFP+ projections, and white asterisks indicate altered organization and fasciculation at the anterior commissure. Below each micrograph, the bright-field images show the general morphology and growth of the corresponding whole animal, with no difference between the three conditions. (**F**) Quantification of the number of EGFP+ neurons in the nasal region of uninjected (open bars), scrmbl MO-injected (solid grey bars) or *z-cb1r* MO-injected (solid black bars) embryos. A significant decrease was observed in embryos injected with the *z-cb1r* MO, compared to control ones. (**G**) Quantification of the misguidance and altered commissural phenotype, expressed as % of the number of embryos observed. A significant increase was observed in embryos injected with the *z-cb1r* MO, compared to control ones. Data are expressed as means ± SEM from three independent experiments. ** = *p* < 0.01; *** = *p* < 0.001. AC, anterior commissure.

First, the efficiency of the *z-cb1r* MO to deplete the endogenous CB1R protein was tested by Western blot analysis on total proteins, extracted from *z-cb1r* MO-injected embryos and from controls (uninjected embryos) at 72 hpf, using an anti-CB1R antibody. A significant dose-dependent reduction of the endogenous level of z-CB1R protein was observed in *z-cb1r* MO-injected embryos, with the most efficient concentration being 400 µM *z-cb1r* MO (Figure 3A). Real-time qPCR analyses on RNA samples extracted from embryos injected with 400 µM *z-cb1r* MO showed that the amount of *z-cb1r* mRNA was reduced in knockdown embryos compared to control uninjected embryos (Figure 3B). The expression levels of three developmental genes, *z-hoxb7a*, *z-hoxa10b* and *z-hox11a* [51], whose expression is age dependent, were quantified by real-time qPCR and employed to exclude any developmental defects of the embryos caused by the injection of *z-cb1r* MO. No significant differences were observed between the *z-cb1r* MO-injected versus the uninjected control embryos (Figure 3B), indicating that the depletion of CB1R did not globally affect the rate of embryonic development.

Having proven the efficiency of *z-cb1r* MO, 1-cell stage *gnrh3::EGFP* embryos were injected with the *z-cb1r* MO at the highest concentration (400 μM) or with scrambled (scrbl)-MO as control, and examined at 72 hpf to determine the number, neurite arborization and commissural organization by confocal microscopy (Figure 3C–E). Upon depletion of CB1R, the number of terminal nerve-associated GnRH3 neurons was significantly reduced in comparison to the controls (average 6 EGFP+ cells in *z-cb1r* MO-treated embryos vs. 10.8 in controls; Figure 3F). We then examined the extension, orientation and fasciculation of EGFP+ fibers in the nasal region and in the basal forebrain. EGFP+ axons displayed an abnormal position, altered direction or aberrant fasciculation within the anterior commissure. We defined as “phenotype” the condition in which we observe a) altered orientation/position of EGFP+ fibers; b) altered fasciculation of EGFP+ fibers; or c) both of these together, as we previously observed with CB1-ligands. The results were then expressed as the number of embryos presenting the phenotype in the various conditions (*z-cb1r* MO *n* = 17; scrbl MO *n* = 17; uninjected *n* = 19) over the total number of embryos examined. As reported in Figure 3G, upon depletion of CB1R, we observed a significant increase in the fraction of embryos with the indicated phenotype compared to the controls (78% in *z-cb1r* MO-injected embryos vs. 14% in uninjected controls).

### 2.4. Expression of CB1R in Developing Zebrafish Brain

**Figure 4:**
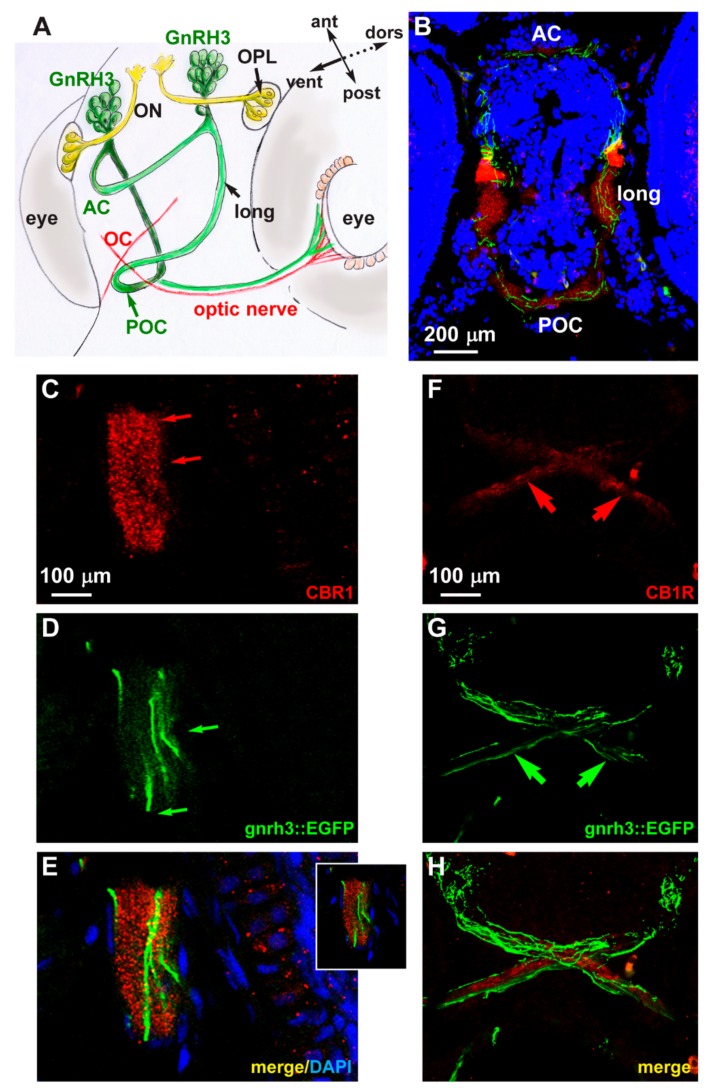
Expression of CB1R in developing zebrafish brain. (**A**) Scheme illustrating the position of the GnRH3 neurons relative to the position and orientation of the main forebrain commissure in the zebrafish brain (redrawn from [52]). (**B**) Low magnification double-fluorescent images of GnRH3::EGFP neurons (green) and IFL with an anti-CB1R antibody (red) on cryostatic sections of the zebrafish head at the age 72 hpf. (**C–E**) Higher magnification double-fluorescence images of the anterior commissure, showing overlapping localization of CB1R punctate staining (red) with EGFP+ fibers (green). Sections were counterstained with DAPI (blue). The merged signal is shown in **E**. Inset in **E** shows a single stack of the same image. Arrows indicate the fluorescence signal. (**F**–**H**) Higher magnification double-fluorescence images of the optic chiasm, immunostained for CB1R (red), as in panels **C**–**E**. Adjacent but non-overlapping expression was observed in these fibers. Arrows indicate the fluorescence signal. Scale bars are reported in panels **B**, in **C** (for **C**,**D**) and in **F** (for **F**–**H**). AC, anterior commissure; ant, anterior; dors, dorsal; long, longitudinal tract; OC, optic chiasm; ON, olfactory nerve; OPL, olfactory placode; POC, postoptic commissure; post, posterior; RGC, retinal ganglion cells; ven, ventral.

We examined the endogenous expression of the CB1 receptor in zebrafish embryos at the age 72 hpf. We carried out fluorescent immunohistochemistry with anti-CB1R antibody on cryostatic sections obtained from *gnrh3::EGFP* embryos. CB1R immunoreactivity (red fluorescence) was detected in various regions of the basal forebrain, in particular in the anterior and postoptic commissures, in the optic chiasm and in the longitudinal tracts. In the same regions, EGFP+ axonal extensions are clearly seen in close proximity to the CB1R signal (Figure 4B). The CB1R immunoreactivity mainly appears as punctate staining (Figure 4C,F). No physical overlap between CB1R immunoreactivity and the GnRH3 axons along the optic tracts and nerves (Figure 4F–H) and no CB1R immunoreactivity in the GnRH3+ cell bodies in the nasal-terminal nerve region were detected (not shown). Colocalization of CB1R immunoreactivity and EGFP fluorescence was instead found in longitudinal fibers and the anterior commissure (Figure 4B–E). These results suggest that CB1R is expressed by GnRH3 fibers in commissures and the longitudinal tracts, while CB1R+ fibers and GnRH3 fibers are mostly distinct, although running in close association along the optic fibers. It should be noted that GnRH3 fibers entering the optic nerve run in an opposite direction to retinofugal CB1R+ axons. Nonetheless, cross talk among GnRH3- and CB1R-containing fibers could occur by means of paracrine and/or cell adhesion signaling.

### 2.5. Effect of Exposure to CB1R Ligands on AgRP1 Neurons

In order to assess possible effects of altered CB1R function on the development of a well-defined group of hypothalamic neurons involved in the control of food-intake, we used a zebrafish strain in which the AgRP1 neurons are genetically marked with mCherry, a red fluorescence reporter [47]. We selected this neuronal population for the following reasons: (1) the neuronal axons and dendrite extend at approximately the same developmental age and for a similar length as the GnRH3 fibers; (2) the AgRP1 axons cross the midline in the anterior and the postoptic commissures, as some of the GnRH3 axons do; and (3) in combination with POMC neurons, the AgRP1 neurons participate in the neuroendocrine control of appetite and energy storage [53]. AgRP1 neurons have cell bodies located in the ventral periventricular hypothalamus with fibers that project towards the rostral, intermediate and dorsal hypothalamus, as well as the preoptic area (POA), the anterior commissure (AC), the post-optic commissure (POC) and the ventral tegmental commissure (VTC) [47].

**Figure 5:**
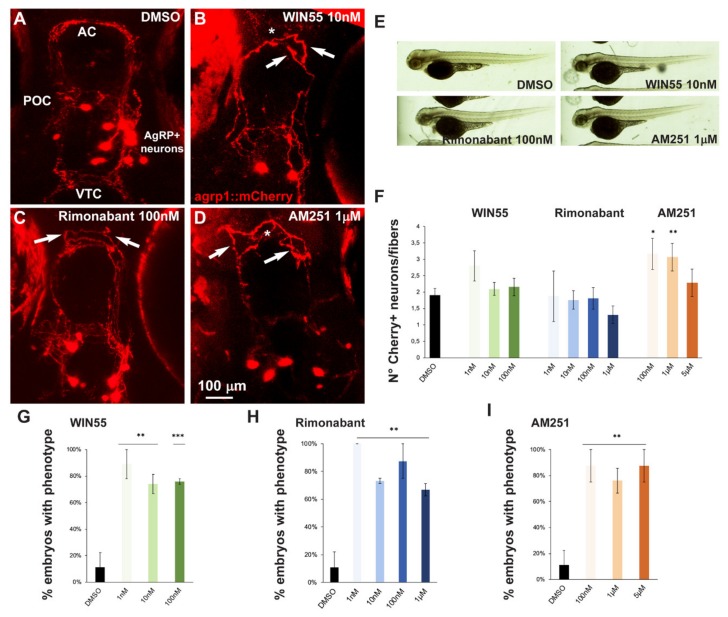
Effect of pharmacological manipulation of CB1R on AgRP1 neurons in zebrafish embryos. (**A–D**) Representative images of mCherry+ neurons and projections in the basal forebrain of embryos, at the age 96 hpf, treated with DMSO only as control (**A**), WIN55 (**B**), Rimonabant (**C**) or AM251 (**D**) at the indicated doses. The observation plane is the same as in Figure 2. Scale bar is reported in (**D**). White asterisks indicate altered fasciculation at the anterior commissure, and white arrows indicate misguided mCherry+ projections. (**E**) Bright-field, low-magnification images of whole fish embryos treated with DMSO only or with the indicated ligands. A normal general morphology and growth was observed. (**F**) Quantification of the ratio of mCherry+ neurons/mCherry+ fibers in embryos treated with DMSO only (solid black bar) or treated with WIN55, Rimonabant or AM251 at the indicated doses. Color code is the same as in Figure 1. Treatment with AM251 resulted in a higher neurons/fibers ratio. (**G–I**) Quantification of the misguidance and altered commissural phenotype, expressed as % of the number of embryos observed upon treatment with WIN55 (**G**), Rimonabant (**H**) or AM251 (**I**). Color code as in Figure 1. Data are expressed as means ± SEM from three independent experiments. * = *p* < 0.05; ** = *p* < 0.01; *** = *p* < 0.001. AC, anterior commissure; POC, postoptic commissure; VTC, ventral tegmental commissure.

For our purposes, we treated embryos derived from breeding between the *agrp1:Gal4* and the *UAS:nfsB-mCherry.* The resultant offspring, *agrp1::mCherry*, were treated with the CB1R ligands WIN55, Rimonabant or AM251 for 96 h as described above. No evident morphological embryos anomalies (Figure 5E) or developmental delays were observed. However, treatment with these compounds resulted in shorter or misoriented mCherry+ fibers, as well as their altered organization at the AC (Figure 5B–D) compared to the control shown in Figure 5A. For quantification purposes we defined as “phenotype” the condition with altered orientation of mCherry+ fibers, or altered fasciculation of mCherry+ fibers, or both of these together. The results were then expressed as the fraction of embryos showing the phenotype over the total number of embryos examined, in percent and reported in Figure 5G–I. The treatment with all three ligands with all doses employed resulted in a significant increase in the fraction of embryos with the indicated phenotype, compared to the controls (Figure 5G–I).

We also determined the number of mCherry+ cells in the treated versus the control embryos, and calculated the ratio between the number of cells and the number of visible fibers; this was done in order to normalize the value between embryos, considering that the absolute number of mCherry+ cells is variable as a consequence of the genetics of the two parent lines [47]. Treatment with AM251, but not with WIN55 or Rimonabant, resulted in a significant increase in the cell number/cell fiber ratio (Figure 5F), indicating a possible reduction in neurite formation.

### 2.6. Search for Genes Functionally Related to CB1R Involved in Axon Fasciculation and Guidance

In order to identify genes related to the CB1R signaling pathway that might participate in axonal growth and fasciculation during development, we resorted to bioinformatic tools and publicly available datasets. First, we selected the following genes known to be involved in axon fasciculation and guidance by a literature search: *ROBO1, PAX6, BRCA1, AKT1, PI3KCA, SLIT2, L1CAM* and *GAP43* [50,54,55,56]. Using these eight genes plus the *CB1R* gene as entry, we searched the Human Protein Reference Database for protein::protein interactions and selected those interactors appearing in the lists of at least two of the entry genes. This search yielded 27 genes (Table S1). Next, we entered 23 of these genes (four were not present in the database) + *CB1R* into the coexpression dataset FuncPred [57], reasoning that genes involved in the same cellular function are significantly coexpressed, and their coexpression is significantly maintained across species [58]. This search yielded a global coexpression network of 625 genes, which was then intersected with a dataset of genes differentially expressed during development of the mouse hypothalamus (Affymetrix MOE430 microarrays, dataset GSE21278 of the GEO repository) [59]. From this last dataset we specifically compared samples of the hypothalamus and POA of C57Bl/6 mouse embryos at E10.5 with the same samples from CD-1 embryos at E18.5, sex-independently. Only genes with *p* < 0.001 and logFC > 1 for upregulated genes, and logFC < −1 for downregulated ones, were considered. This intersection yielded a total of 269 “best candidates”, 207 of which were upregulated and 62 of which were downregulated during hypothalamus development (Table S2). Candidates resulting from this intersection were then functionally annotated and the following relevant over-represented categories were detected: “cytoskeleton”, 13%; “synapse”, 12%; “neuron projection”, 10%.

Among the genes appearing in relevant functional classes we noted the presence of semaphorins, cell adhesion molecules, kinesins, GABA and glutamate receptors. Finally, from the list of relevant genes we further selected them by a literature search and came up with a set of 8 genes putatively interacting with CB1R, or sharing a common expression pattern, and potentially involved in axon elongation–fasciculation-guidance: Glycoprotein M6A *(GPM6A)*, N-deacetylase and N-sulfotransferase-3 *(NDST3,* also known as *HSST3),* Neural growth regulator-1(*NEGR1),* Sodium voltage-gated channel alpha subunit-2 *(SCN2A,* also known as *Na(v)1.2),* Seizure related gene-6 (*SEZ6),* HECT, C2 and WW domain containing E3 ubiquitin protein ligase-1 *(HECW1,* also known as *NEDL1),* Septin-6 *(SEPT6)* and Stathmin-2 *(STMN2,* also known as *SCG10).*

For further analyses, we focused on *z-stmn2a, z-stmn2b, negr1, z-sez6a* and *z-sez6b*, since they have been reported as regulator of axons growth and cell migration [60,61,62,63]. We decided to define whether the expression of these selected candidates changes following treatment with the CB1R ligands WIN55, Rimonabant and AM251, or following depletion of endogenous CB1R by MO injection. 

**Figure 6:**
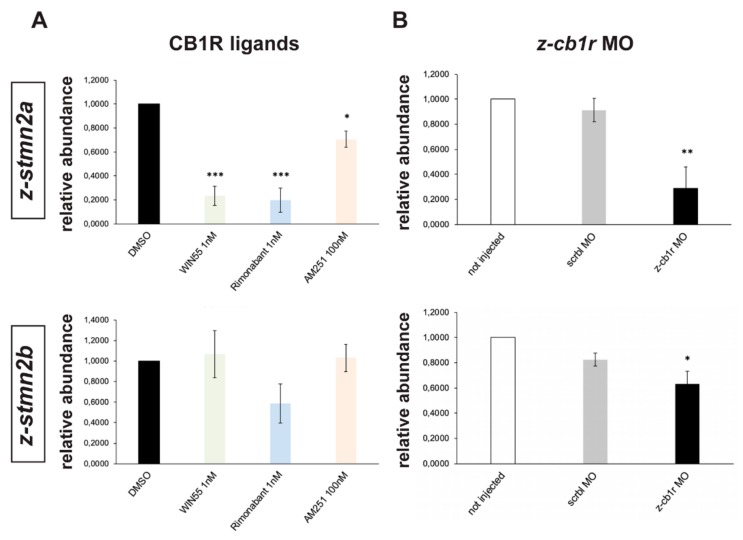
Expression of *z-stmn2a* and *z-stmn2b* upon treatment with CB1R ligands or CB1R knockdown in early zebrafish embryos. (**A**) Real-time qPCR analyses of *z-stmn2a* (top histogram) and *z-stmn2b* (bottom histogram) on RNA samples obtained from embryos treated with the indicated CB1R ligands, at the indicated doses. Expression is shown relative to the expression of the housekeeping mRNA (*z-β-actin*). The abundance of the control RNA (DMSO) was set to 1. A reduced abundance of *z-stmn2a* mRNA was observed in embryos treated with the ligands, as compared to embryos treated with DMSO only. (**B**) Same analysis as above, on RNA samples obtained from uninjected (open bars), scrambled MO-injected (solid grey bars) or *z-cb1r* MO-injected (solid black bars) embryos. A reduced expression of *z-stmn2a* and *z-stmn2b* mRNAs was observed in embryos injected with *z-cb1r* MO as compared to the controls. * = *p* < 0.05; ** = *p* < 0.01; *** = *p*<0.001.

Real-time qPCR was used to compare the abundance of *z-stmn2a*, *z-stmn2b*, *negr1*, *z-sez6a* and *z-sez6b* mRNAs in samples extracted from embryos treated with WIN55 (1 nM), Rimonabant (1 nM) or AM251 (100 nM), or treated with DMSO only as control. All treatments resulted in a significant reduction in the abundance of *z-stmn2a* relative to the control (Figure 6A), while the expression of *z-stmn2b* (Figure 6A) and of the other targets (data not shown) did not significantly change.

Similarly, expression levels of *z-stmn2a* and *z-stmn2b* was analyzed in *z-cb1r* MO, scrbl MO or uninjected zebrafish embryos. In CB1R-depleted embryos we observed a significant reduction in the abundance of *z-stmn2a* and *z-stmn2b* relative to the controls (Figure 6B), while the expression of the other candidates did not change (data not shown).

In conclusion, the expression of the *z-stmn2a* gene in zebrafish embryos is consistently modulated by treatments reducing or interfering with the CB1 receptor.

## 3. Discussion

In the present paper we show that pharmacological manipulation and gene knockdown of CB1R in early zebrafish embryos cause profound alterations in the ability of GnRH3 and AgRP1 axons to organize in discrete and well-fasciculated commissures, accompanied by misrouting and an altered neuron number. We also provide protein coexpression analyses that confirm, in early embryos, the localization of CB1 receptors in commissural structures and longitudinal tracts associated with GnRH3 fibers. Thus, we propose that CB1R is involved in the fasciculation and guidance of neuroendocrine GnRH3 and AgRP1 axons during forebrain development, implying high sensitivity of these neuroendocrine systems to neurodevelopmental exposure to exogenous cannabinoids.

During brain development, axonal projections elongate, fasciculate and defasciculate in distinctive pathways, often crossing the midline and reaching the appropriate targets guided by the orchestrated interactions between axon tracts and the environment at distinct domains, as well as homo- and hetero-philic interactions among axonal fibers. Several signaling and adhesion molecules involved in axon guidance and fasciculation have been identified [64,65]. Recent reports suggest that CB1R signaling is likely to modulate one or more of these signaling cascades during neural circuit formation, however most studies have focused on a few brain regions and neuronal types, in particular corticofugal and retinofugal neurons [22,23,24,25,66,67,68,69]. Here we show a novel role of eCBs signaling via CB1R on the development of two well-characterized neuroendocrine systems in zebrafish, the GnRH3 hypophysiotropic system and the AgRP1 system, involved in the central control of reproduction and food intake, respectively.

In order to gain relevant information on the developmental effects of the synthetic CB1R ligands employed in this study (WIN55, Rimonabant and AM251) we tested the possible toxic effects of their chronic exposure on zebrafish embryos from 0 to 72 or 96 hpf using a wide range of concentrations. Embryo survival was affected only by the highest agonist concentration (1 μM WIN55), and therefore this concentration was excluded from subsequent experiments. The selected ligands concentrations did not interfere with the overall embryo development and the hatching rate. These results partly agree with a previous study [70], in which AM251 treatment (10 and 20 nM) on zebrafish embryos for 72–96 hpf did not lead to morphological changes but decreased the hatching rate.

We analyzed the impact of chronic CB1R ligand exposure on the developing GnRH3 and AgRP1 systems, taking advantage of the fluorescently labelled neurons present in the *gnrh3::EGFP* and the *agrp1::mCherry* strains [47,71]. Pharmacological interference with CB1R in *gnrh3::EGFP* embryos clearly showed abnormal fasciculation of GnRH3 axons in the anterior commissure, with some ectopic axons detaching from the commissure itself. Defective phenotypes resulted from exposures to all three tested drugs (CB1R agonist, inverse agonist and antagonist) even at the lowest concentrations, indicating that interfering with the endogenous eCB system, either through over-activation or inhibition, equally disrupts the modulatory control exerted by CB1R on axonal navigation and fasciculation. We obtained similar results by knocking down the CB1R with a morpholino strategy, confirming previous observations made on the anterior commissure [30]. Furthermore, by immunocytochemistry we show co-expression of CB1R and GnRH3::EGFP in several fiber systems, although evidence of co-localization of the two labeling in exactly the same axons was found only in the anterior commissure and in longitudinal fiber tracts. Together these data suggest that at least part of the axons crossing the anterior commissure are affected by CB1R ligand exposure, including CB1R-expressing GnRH3 fibers.

In addition to the defects observed in fiber tracts, we found a significant decrease in the number of GnRH3 cell bodies (terminal nerve-associated GnRH3 neurons) following both antagonist (AM251) exposure and CB1R knockdown. These results are consistent with the previously reported role of CB1R as a regulator of progenitor niches [7,23]. Alternatively, CB1R could be involved in the timing of GnRH3 neuronal migration. A role of CB1R in neuronal migration has been previously reported during radial migration of immature pyramidal neurons in the cortex [23,72], and eCB signaling has been shown to promote migration of newborn neurons along the rostral migratory stream in the postnatal mouse brain [14]. Further experiments are needed to define a possible role of CB1R in GnRH3 neuronal migration.

Pharmacological interference of CB1R in *agrp1::mCherry* zebrafish embryos resulted in anterior commissure defects similar to those observed in *gnrh3::EGFP* embryos, suggesting that a common mechanism affected both GnRH3- and AgRP1-containing fibers. We found an increase in the number of AgRP1+ cell bodies in the hypothalamus in AM251-treated embryos, however this result could be affected by the methodology we chose for counting cells. It is possible that the apparent augmented number of AgRP1 cells depends on a decrease in fiber number, which would be in line with an axonal defect. 

In our experiments, we observed similar phenotypes when applying CB1R agonists or antagonist/inverse agonists. This is consistent with the fact that different studies reported opposite effects of CB1R activation on axonal growth. Indeed, in some cases CB1R-activation has been reported to induce collapse of the growth cone and axon retraction, while its inhibition resulted in axon elongation [9,10,22,25,49]; other studies reported the opposite effects [23,73]. It should be kept in mind that while eCBs can reach very high local concentrations due to spatially and temporally coordinated activity of their synthetic and degrading enzymes, exogenous CB1R agonists and antagonists engage their targets indiscriminately, possibly impacting the spatial- and chrono-dynamics of eCB signaling in developing axons in a similar fashion.

Multiple mechanisms downstream of CB1R activation in developing neurons have been suggested. It has been reported that CB1R acts via the WAVE complex and Rac1-GTPases, and via Rho-GTPase and the Rho-dependent ROCK activation [9,10]; these pathways appear to mainly control the dynamics of the actin cytoskeleton. Notably, these intracellular signaling pathways are essential for neuronal “motility” during embryo development, including migration, neuritogenesis, spinogenesis and synaptogenesis [74].

Another reported mechanism involves the stathmin family of proteins and the fine modulation of microtubule dynamics at the growth cone. Stathmin-2, also known as SCG10 (superior cervical ganglia neural-specific 10) is a neuron-specific protein highly expressed in the developing forebrain, able to stabilize the plus ends both at steady state and early during polymerization by increasing the rate and extent of growth and facilitating microtubule extension into filopodia [13]. Overexpression of stathmin-2 strongly enhances neurite outgrowth, while its phosphorylation by the JNKs negatively regulates its microtubule destabilizing activity, suggesting that the JNK-stathmin-2 pathway may link extracellular signals to the rearrangement of the neuronal cytoskeleton [11,12]. By applying a bioinformatic analysis based on conserved coexpression and transcriptomic datasets from the developing hypothalamus, we report the identification of stathmin-2 as most likely to be involved in the effect of CB1R on axon elongation/fasciculation/guidance. Recently, stathmin-2 has been shown to be a specific molecular target for a CB1R-mediated effect of ∆9-THC in mouse and possibly in human fetal nervous systems [29]. By restoring stathmin-2 expression in the context of ∆9-THC exposure and consequent CB1R activation, the authors were able to acutely correct the altered cytoskeleton dynamics [29]. Highly relevant for neuroendocrine neurons, stathmin expression has been shown to modulate the migratory properties of mammalian GnRH neurons in vitro [60]. Thus, several evidence including ours indicate that stathmin-2/SCG10 participates in a regulatory cascade that is influenced by the activity and/or misactivity of the CB1R and controls cytoskeleton dynamics during neurite elongation and possibly neuronal migration.

## 4. Materials and Methods

### 4.1. Zebrafish Strains and Treatments 

All procedures using zebrafish (*Danio rerio*) were authorized by the Ethical Committee of the University of Torino and the Italian Ministry of Health (approval code: N. 425/2016-PR; 27 April 2016). The wild-type fish strain TL-AB was used. The fish strain Tg(*gnrh3::EGFP*) [45,46,71] was obtained from Prof. Y. Zohar (Univ. Maryland Biotechnology Institute, Baltimore, MD, USA). The fish strain named, for simplicity, *agrp1::mCherry* was obtained by breeding the *TgBAC(agrp:GAL4-VP16)* with the *UAS::nfsB-mCherry* strains, followed by selection of the reporter fishes by quick fluorescent examination at low magnification [47]. Adult fish were routinely maintained under a 14 h light and 10 h dark photoperiod at approximately 28 °C, bred and genotyped according to standard procedures. Allelic transmission followed the expected mendelian ratios. Eggs were generated by natural mating, and following fertilization were collected, treated and maintained under a 12 h light and 12 h dark photoperiod, incubated at 28 °C.

The *Tg(gnrh3::EGFP)* embryos were grown in the presence of 0.003% 1-phenyl-2-thiourea (PTU) to prevent the formation of melanin pigment, which could interfere with the visualization of fluorescence in neurons and fibers; the *agrp1::mCherry* embryos were grown in fish water without PTU, because the pigmentation in this case did not affect the analysis under microscope.

### 4.2. Evaluation of Survival Rate and Hatching Rate

The endpoints used to assess developmental toxicity comprised embryos survival (percentage of live embryos/total) and hatching rate (percentage of hatching embryos/living embryos), observed every 24 h during the whole exposure period (72 or 96 hpf). Dead embryos were removed without solution renewal.

### 4.3. Morpholino Injections

To down-modulate CB1R we utilized a conventional antisense morpholino oligonucleotide (MO)-mediated strategy (GeneTools, LLC, Philomat, OR, USA). The *z-cb1r* MO was designed as previously published [30]. The MOs used for injection were
*z-cb1r* MO:5′ CTAGAGGAAACCTGTCGGAGGAAAT 3′Mismatch_PPFIA scrambled MO (control):5′ TCGTGGCCATCAACTCGAACA 3′.

Zygotes were collected at the 1-cell stage and injected under stereological examination with 50 µM, 200 µM or 400 µM of *z-cb1r* MO (or the scrambled MO as control), in the presence of Phenol Red for subsequent selection of the injected embryos. Uninjected embryos were also analyzed.

### 4.4. Western Blot

Zebrafish embryonic heads at 72 hpf were lysed manually in modified RIPA buffer composed as follows: 150 mM NaCl, 50 mM Tris pH 7.5 with 1% Triton X-100, 0.5% Na deoxycholate, 0.1% SDS, 2 mM EDTA, 1 mM sodium orthovanadate, 1 mM sodium fluoride and protease inhibitors. Homogenates were centrifuged at 3000 rpm for 5 min at 4 °C, extracts were quantified with BCA method (Bradford). A total of 30 µg of proteins/lane were separated in 10% acrylamide gel and transferred to a PVDF membrane (Millipore, Burlington, MA, USA). Membranes were saturated with 5% BSA in 0.3% TBS-Tween-20, washed in TBS-Tween, incubated overnight at 4 °C with an anti-CB1R primary antibody (diluted 1:800), then washed in TBS-Tween, incubated with an anti-rabbit IgG HRP-conjugated secondary antibody (diluted 1:3000; BioRad, Hercules, CA, USA), washed and developed with chemo-luminescence reagent Luminata TM Forte Western HRP Substrate (Millipore). Images were acquired with the ChemiDoc system (Bio-Rad), exported and analyzed.

### 4.5. Immunostaining

*gnrh3::EGFP* fish embryos were collected at 72 hpf and processed as described above. Embryos were sacrificed with a tricaine overdose, fixed in 4% PFA overnight, then washed in PBS. Solution was replaced with 0.1M PB and 7% sucrose at 4 °C for 12 h, then with 0.1M PB and 30% sucrose at 4 °C overnight. Samples were then embedded in OCT (Bio Optica, Mila, Italy) and sectioned at 14 μm thickness. Sections were washed in 0.01M PBS pH 7.4 for 10 min and incubated in normal goat serum (diluted 1:100 in PBS-Triton-X 100), then incubated with the anti-CB1R antibody (diluted 1:600 in PBS-Triton-X 100-normal goat serum) O/N. Slices were then washed in 0.01M PBS pH 7.4 for 10 min and incubated with anti-rabbit IgG CY3-conjugated secondary antibody (diluted 1:800 in PBS-Triton-X 100; Jackson ImmunoResearch Laboratories, Philadelphia, PA, USA) for 45 min. For cellular nuclei visualization, slices were incubated with DAPI (4′,6-diamidine-2′-phenylindole dihydrochloride), then washed in PBS and mounted in DABCO. The fluorescent signal was visualized using a Leica TCS SP5 (Leica Microsystem, Wetzlar, Germany).

### 4.6. Bioinformatic Searches and Statistical Analyses

Known and putative protein::protein interactions were searched for using the Human Protein Reference Database (http://www.hprd.org). Only those which appear in the list of at least two entry genes were further considered. Conserved coexpression in transcriptomic datasets was searched for using FuncPred (https://www.mbc.unito.it/en/notizie/funcpred) [57,58]. Conversion of the “gene ID” from human to mouse was done using the BioMart tool (http://www.biomart.org).

As a reference dataset of differentially expressed genes in the hypothalamus, we used the dataset GSE21278 present in Gene Expression Omnibus (http://www.ncbi.nlm.nih.gov/geo/) [59]. This data set provides extensive gene expression data on developing mouse hypothalamic tissues, done using the Affymetrix MOE430 microarray. 

Functional annotation and definition of the over-represented categories was done using the tools available at http://geneontology.org/page/go-enrichment-analysis and at http://amp.pharm.mssm.edu/Enrichr/.

All data were statistically evaluated using commercially available software (SPSS version 18.0 for Windows, SPSS Inc., Chicago, IL, USA; and Microsoft Excel 2011, Microsoft Corporation, Albuquerque, NM, USA). Significance was calculated using a t-test and one-way ANOVA followed by Bonferroni’s post hoc test for multiple comparisons.

### 4.7. Real-Time qPCR Analysis

Total RNA was extracted from 72 hpf embryo heads with the TRIzol reagent (Life Technologies, Carlsbad, CA, USA), following the manufacturer’s instructions. Genomic DNA was destroyed by passing each sample into a 22G needle connected to a 1 mL syringe. Chloroform was added and, after 15–20 min of centrifugation at 12,000× *g* at 4 °C, RNA was precipitated in isopropanol. Samples were centrifuged at 12,000× *g* at 4 °C for 15 min and the RNA was then washed with 75% ethanol. The RNA pellet was briefly air-dried, resuspended in 20 µL sterile water and stored at −20 °C. Samples were quantified using a NanoDrop1000 spectrophotometer (Nanodrop Technologies, Inc., Wilmington, DE, USA). Real-time qPCR was performed using Superscript III Platinum One-step qRT-PCR system (Invitrogen, Carlsbad, CA, USA) and the thermal cycler Rotor Gene Q (Qiagen, Hilden, Germany). Each RNA sample was analyzed in three technical replicates containing 50 ng of total RNA. Primers sequences were
*z-β-actin*FCCCACATAGGAGTCTTTCTG
RTCCCCTTGTTCACAATAACC*z-cb1r*FCAGAACAGATCATACACCATGA
RTGGTCTTATTCATCATCTACGC*z-hoxb7a*FGCTGGCGATCTCTGTAAAGC
RTTTTGATGGTAGCCCCTCTG*z-hoxa10b*FGAGCTAAGGGGGTCCACTG
RCACTTTTGGAATCTCCTGCTTT*z-hox11a*FCAGCAAACAGTCGACACCAC
RCGGTCGCTCCTTTCCTTC*z-stmn2a*FTCCATGCTCTCGCTTATTTG
RGGAGGATGTAGAGATGTGCT*z-stmn2b*FATGGAGCAGATCAAGGAGAA
RGGAGGATGTAGAGATGTGCT*z-sez6a*FAGTTTAGCAGTGAACGAACC
RGAAGACCAGTGACTGAAACC*z-sez6b*FGTCCATTTTCATCCCTGTGG
RGGCTGCTTTGAATCATAGGT*z-negr1*FTTTTCGAGTGGTACAAGGGA
RGGGTTGGATGGATTCAGAGG

Quantification of mRNA abundance in each sample was done using a standard curve, built with several dilution of the samples. The abundance of the *z-β-actin* housekeeping mRNA was used for normalization. The mRNA expression was calculated relative to the one of control embryos (=1).

### 4.8. CB1 Ligands Exposure and Confocal Microscope Analysis

Upon fertilization, at the 1-cell stage, embryos were treated with the following ligands (Tocris Bioscience, Bristol, UK): CB1R-agonist WIN 55,212-2 (renamed WIN55 for simplicity, 1 nM, 10 nM and 100 nM); CB1R-inverse agonist Rimonabant (SR141716A) (1 nM, 10 nM, 100 nM and 1 μM); CB1R-antagonist AM251 (100 nM, 1 μM and 5 μM). All these compounds were dissolved in 0.1% dimethyl-sulfoxide (DMSO) and added to the fish water for 72 h (*gnrh3::EGFP*) or 96 h (*agrp1::mCherry*) without solution replacement for the entire exposure period. At 24 hpf 0.003% PTU was added to the water for *gnrh3::EGFP* fish embryos. Embryos were grown in 6-well Petri dishes, 30 embryos/well. Negative control embryos were treated with 0.1% DMSO alone. 

For whole mount visualization, the *gnrh3::EGFP* and *agrp1::mCherry* embryos were collected at 72 hpf or 96 hpf, sacrificed with a tricaine overdose, fixed with 4% paraformaldehyde (PFA) at 4 °C O/N, washed in PBS and embedded in 4% low melting agarose. The apical portion of the head was manually dissected from the rest of the embryo. Confocal microscopy examination was carried out at 20× and 40× magnification with a Leica TCS SP5 or Leica TCS SP8 (Leica Microsystems). Images were acquired as Z-stacks of 5 μm-thick optical sections. For counting, stacks of Z-slices at 40× were used, images were processed and analyzed with the assistance of ImageJ software (NIH, Bethesda, MD, USA) after adjustment of contrast and brightness.

### 4.9. Statistical Analyses

Data are expressed as mean ± standard error of the mean (SEM) from three independent experiments. The results of pharmacological treatments/morpholino injections were compared to control (DMSO-treated/uninjected embryos). Statistical analyses were performed using commercially available software (SPSS version 18.0 for Windows and Microsoft Excel 2011). Significance was calculated using Fisher’s exact test for the analysis of altered phenotypes and one-way analysis of variance (ANOVA) for all other experiments, followed by Bonferroni’s and Tuckey’s post hoc tests for multiple comparisons. *p* ≤ 0.05 was taken as minimum level of significance. 

## 5. Conclusions

The impact of exogenous cannabinoids on the gonadotropic neurons and the orexigenic/anorexigenic neurons has long been investigated [36,75,76]. Most previous work has focused on the adolescent or adult brain, while the effect of interfering with CB1R in early development of neuroendocrine neurons has remained poorly investigated. Based on our results, exposure to low doses of CB1R ligands during critical phases of development could alter axon pathfinding of GnRH3 and AgRP1 neurons, and possibly other neurons, potentially leading to a range of long-term chronic deficits of sexual maturation/reproduction and/or in the control of food intake/energy accumulation. Importantly, the hypothalamic neurons attaining the control of sexual maturation/reproduction and the control of food intake, are synaptically and functionally linked, as indicated by the fact that alterations of food intake control often lead to sexual disturbances, in mice as well as in humans [44]. These issues deserve further studies in other animal models, in order to attest the realistic possibility that embryo exposure to exogenous cannabinoid ligands could impact on the development of GnRH and AgRP neuroendocrine systems.

## Supplementary Materials

Supplementary Materials can be found at https://www.mdpi.com/1422-0067/21/1/168/s1.

## Abbreviations

∆9-THC	Δ9-tetrahydrocannabinol
2 AG	2-arachidonoylglycerol
AC	anterior commissure
AgRP	agouti-related peptide
AgRP1	agouti-related peptide 1
AM251	(*N*-(Piperidin-1-yl)-5-(4-iodophenyl)-1-(2,4-dichlorophenyl)-4-methyl-1*H*-pyrazole-3-carboxamide)
CB1R	cannabinoid receptor type 1
CB2R	cannabinoid receptor type 2
CBD	cannabidiol
DAGL	diacylglycerol lipase
DMSO	dimethyl sulfoxide
dpf	days post fertilization
eCB	endocannabinoid
GnRH	gonadotropin-releasing hormone
GTPase	guanosine triphosphatase
Hox	homeobox gene
hpf	hours post fertilization
JNK1	c-Jun N-terminal kinase 1
MAGL	monoacylglycerol lipase
MO	morpholino oligonucleotide
Negr1	neuronal growth regulator 1
POA	pre-optic area
POC	post-optic commissure
Rimonabant	SR141716, (*N*-(Piperidin-1-yl)-5-(4-chlorophenyl)-1-(2,4-dichlorophenyl)4-methyl-1*H*-pyrazole-3-carboxamide hydrochloride)
Sez6	Seizure protein 6
Stmn2/SCG10	Stathmin2/Superior Cervical Ganglion-10 Protein
VTC	ventral tegmental commissure
WIN55	WIN 55,212-2; (*R*)-( + )-[2,3-Dihydro-5-methyl-3-(4-morpholinylmethyl)pyrrolo [1,2,3-*de*]-1,4-benzoxazin-6-yl]-1-naphthalenylmethanone mesylate
