# Supplementary material for: Interference with the Cannabinoid Receptor CB1R Results in Miswiring of GnRH3 and AgRP1 Axons in Zebrafish Embryos
Source: Int J Mol Sci. 2019 Dec 25;21(1):168. doi: 10.3390/ijms21010168 (PMC6982252; doi:10.3390/ijms21010168)
Supplement: Supplementary file 1 [file ijms-21-00168-s001.zip › Suppl Table I.docx]

**Supplementary Table I.**

Protein::protein interactors in common with CB1r and selected proteins involved in axon guidance and fasciculation.

| **Gene ID** | **Gene Name** |
| --- | --- |
| 25 | ABL |
| 207 | AKT1 |
| 334 | Amyloid precursor like protein 2 |
| 367 | Androgen receptor |
| 672 | BRCA1 |
| 863 | Caspase 3, apoptosis-related cysteine peptidase |
| 1268 | cnr1 |
| 1457 | Casein kinase II, alpha 1 |
| 2033 | E1A binding protein p300 |
| 2099 | Estrogen receptor alpha |
| 2175 | Fanconi anemia, complementation group A |
| 2596 | GAP43 |
| 3897 | L1CAM |
| 4088 | SMAD family member 3 |
| 4089 | SMAD4 |
| 5080 | PAX6 |
| 5290 | PIK3CA |
| 5295 | Phosphatidylinositol 3 kinase regulatory subunit, alpha |
| 5580 | Protein kinase C delta |
| 6091 | Robo1 |
| 6092 | Hypothetical protein KIAA1568 (known as ROBO2) |
| 6198 | Ribosomal protein S6 kinase |
| 6597 | SMARCA4 |
| 6772 | STAT1 |
| 8682 | PEA15 |
| 9353 | Slit2 |
| 26060 | AKT2 interactor |
