# Supplementary material for: Interference with the Cannabinoid Receptor CB1R Results in Miswiring of GnRH3 and AgRP1 Axons in Zebrafish Embryos
Source: Int J Mol Sci. 2019 Dec 25;21(1):168. doi: 10.3390/ijms21010168 (PMC6982252; doi:10.3390/ijms21010168)
Supplement: Supplementary file 1 [file ijms-21-00168-s001.zip › Suppl Table II.docx]

**Supplementary Table II.**

Genes resulting from intersection between the network of conserved coexpressed genes reported in

Supplementary Table I with the transcriptome of the developing mouse hypothalamus.

**Upregulated**

| **Gene.title** | **Gene.ID** |
| --- | --- |
| megalencephalic leukoencephalopathy with subcortical cysts 1 homolog (human) | 170790 |
| neuronal growth regulator 1 | 320840 |
| CUGBP, Elav-like family member 2 | 14007 |
| myelin transcription factor 1-like | 17933 |
| glycoprotein m6a | 234267 |
| RUN and SH3 domain containing 1 | 72296 |
| neurotrophic tyrosine kinase, receptor, type 2 | 18212 |
| RAB6B, member RAS oncogene family | 270192 |
| glutamate receptor, ionotropic, AMPA2 (alpha 2) | 14800 |
| cell adhesion molecule with homology to L1CAM | 12661 |
| single-stranded DNA binding protein 2 | 66970 |
| beta-1,3-glucuronyltransferase 1 (glucuronosyltransferase P) | 76898 |
| seizure related gene 6 | 20370 |
| islet cell autoantigen 1-like | 70375 |
| solute carrier family 12, member 5 | 57138 |
| chloride channel 3 | 12725 |
| protein kinase inhibitor, alpha | 18767 |
| sodium channel, voltage-gated, type II, alpha 1 | 110876 |
| RIKEN cDNA 2010107G23 gene | 69894 |
| glycoprotein m6b | 14758 |
| secretory carrier membrane protein 1 | 107767 |
| prion protein | 19122 |
| ankyrin repeat and sterile alpha motif domain containing 1B | 77531 |
| calcium/calmodulin-dependent protein kinase IV | 12326 |
| leucine rich repeat transmembrane neuronal 2 | 107065 |
| gamma-aminobutyric acid (GABA) A receptor, subunit beta 3 | 14402 |
| ArfGAP with RhoGAP domain, ankyrin repeat and PH domain 2 | 212285 |
| calmodulin 1 | 12313 |
| latrophilin 3 | 319387 |
| neural precursor cell expressed, developmentally down-regulated gene 4-like | 83814 |
| glycerophosphodiester phosphodiesterase domain containing 1 | 66569 |
| atlastin GTPase 1 | 73991 |
| ring finger protein 14 | 56736 |
| kinase D-interacting substrate 220 | 77480 |
| BR serine/threonine kinase 2 | 75770 |
| calcium channel, voltage-dependent, N type, alpha 1B subunit | 12287 |
| protocadherin 7 | 54216 |
| dimethylarginine dimethylaminohydrolase 1 | 69219 |
| synaptotagmin IV | 20983 |
| septin 3 | 24050 |
| contactin 1 | 12805 |
| serine/arginine-rich splicing factor 12 | 272009 |
| leucine rich repeat protein 3, neuronal | 16981 |
| solute carrier family 1 (glial high affinity glutamate transporter), member 3 | 20512 |
| cadherin, EGF LAG seven-pass G-type receptor 3 (flamingo homolog, Drosophila) | 107934 |
| kinesin light chain 1 | 16593 |
| calmodulin regulated spectrin-associated protein family, member 3 | 69697 |
| FXYD domain-containing ion transport regulator 6 | 59095 |
| catenin (cadherin associated protein), alpha 2 | 12386 |
| calcium/calmodulin-dependent protein kinase II, delta | 108058 |
| lipoma HMGIC fusion partner-like protein 4 | 269788 |
| microtubule-associated protein 2 | 17756 |
| HECT, C2 and WW domain containing E3 ubiquitin protein ligase 1 | 94253 |
| ATPase, aminophospholipid transporter-like, class I, type 8A, member 2 | 50769 |
| ELAV (embryonic lethal, abnormal vision, Drosophila)-like 2 (Hu antigen B) | 15569 |
| neural cell adhesion molecule 1 | 17967 |
| Abelson helper integration site 1 | 52906 |
| cyclin-dependent kinase 5, regulatory subunit 1 (p35) | 12569 |
| metallothionein 3 | 17751 |
| ATPase, H+ transporting, lysosomal V0 subunit A1 | 11975 |
| neuron specific gene family member 2 | 18197 |
| protocadherin alpha subfamily C, 2///protocadherin alpha subfamily C, 1///protocadherin alpha 8///protocadherin alpha 2///protocadherin alpha 12///protocadherin alpha 3///protocadherin alpha 9///protocadherin alpha 1///protocadherin alpha 10///protocadherin alpha 11///protocadherin alpha 5///protocadherin alpha 7///protocadherin alpha 6///protocadherin alpha 4 | 353237///353236///353235///353234///192164///192163///192161///116731///12943///12942///12941///12939///12937///12936 |
| secernin 1 | 69938 |
| WD repeat and SOCS box-containing 2 | 59043 |
| guanine nucleotide binding protein, alpha q polypeptide | 14682 |
| ganglioside-induced differentiation-associated protein 1-like 1 | 228858 |
| glutamate receptor, ionotropic, kainate 2 (beta 2) | 14806 |
| protocadherin 9 | 211712 |
| prolyl endopeptidase-like | 213760 |
| 1-acylglycerol-3-phosphate O-acyltransferase 3 | 28169 |
| aquaporin 4 | 11829 |
| gamma-aminobutyric acid (GABA) A receptor, subunit alpha 2 | 14395 |
| TAO kinase 3 | 330177 |
| sodium channel, voltage-gated, type I, alpha | 20265 |
| endonuclease domain containing 1 | 71946 |
| SOGA family member 3 | 67412 |
| tankyrase, TRF1-interacting ankyrin-related ADP-ribose polymerase 2 | 74493 |
| islet cell autoantigen 1 | 15893 |
| family with sequence similarity 63, member B | 235461 |
| transmembrane protein 35 | 67564 |
| microtubule-associated protein, RP/EB family, member 2 | 212307 |
| transmembrane protein 30A | 69981 |
| tripartite motif-containing 2 | 80890 |
| potassium voltage-gated channel, subfamily Q, member 2 | 16536 |
| cytoplasmic polyadenylation element binding protein 4 | 67579 |
| microtubule associated serine/threonine kinase 1 | 56527 |
| calbindin 1 | 12307 |
| runt-related transcription factor 1; translocated to, 1 (cyclin D-related) | 12395 |
| adrenergic receptor kinase, beta 2 | 320129 |
| synaptotagmin VII | 54525 |
| collapsin response mediator protein 1 | 12933 |
| cancer susceptibility candidate 4 | 319996 |
| microtubule-associated protein, RP/EB family, member 3 | 100732 |
| kinesin family member 5C | 16574 |
| zinc finger, DHHC domain containing 23 | 332175 |
| internexin neuronal intermediate filament protein, alpha | 226180 |
| nuclear factor I/A | 18027 |
| aldolase C, fructose-bisphosphate | 11676 |
| tissue inhibitor of metalloproteinase 2 | 21858 |
| cyclic AMP-regulated phosphoprotein, 21 | 74100 |
| f-box protein 9 | 71538 |
| nuclear factor I/B | 18028 |
| reticulon 3 | 20168 |
| synaptogyrin 3 | 20974 |
| DDHD domain containing 1 | 114874 |
| synaptoporin | 72003 |
| APC membrane recruitment 3 | 211383 |
| Eph receptor A7 | 13841 |
| SNRPN upstream reading frame///small nuclear ribonucleoprotein N | 84704///20646 |
| nuclear receptor subfamily 3, group C, member 1 | 14815 |
| ATPase, Ca++ transporting, cardiac muscle, slow twitch 2 | 11938 |
| CD47 antigen (Rh-related antigen, integrin-associated signal transducer) | 16423 |
| chondroitin sulfate N-acetylgalactosaminyltransferase 1 | 234356 |
| double C2, alpha | 13446 |
| clusterin | 12759 |
| limb region 1 | 56873 |
| deformed epidermal autoregulatory factor 1 (Drosophila) | 54006 |
| stathmin-like 3 | 20262 |
| reticulon 2 (Z-band associated protein) | 20167 |
| solute carrier family 23 (nucleobase transporters), member 2 | 54338 |
| CD200 antigen | 17470 |
| serine/arginine-rich protein specific kinase 2 | 20817 |
| guanine nucleotide binding protein (G protein), gamma 3 | 14704 |
| myeloid/lymphoid or mixed-lineage leukemia (trithorax homolog, Drosophila); translocated to, 11 | 56772 |
| vacuolar protein sorting 26 homolog B (yeast) | 69091 |
| centrosomal protein 170 | 545389 |
| RAB11A, member RAS oncogene family | 53869 |
| WNK lysine deficient protein kinase 2 | 75607 |
| microtubule-associated protein 1B | 17755 |
| RAB39B, member RAS oncogene family | 67790 |
| septin 6 | 56526 |
| SUB1 homolog (S. cerevisiae) | 20024 |
| Kruppel-like factor 12 | 16597 |
| nuclear undecaprenyl pyrophosphate synthase 1 homolog (S. cerevisiae) | 52014 |
| family with sequence similarity 49, member A | 76820 |
| CLIP associating protein 2 | 76499 |
| thymoma viral proto-oncogene 3 | 23797 |
| v-ral simian leukemia viral oncogene homolog A (ras related) | 56044 |
| hepatocyte cell adhesion molecule | 72927 |
| progestin and adipoQ receptor family member VIII | 74229 |
| unc-5 homolog D (C. elegans) | 210801 |
| DnaJ (Hsp40) homolog, subfamily A, member 4 | 58233 |
| Ras association (RalGDS/AF-6) and pleckstrin homology domains 1 | 77300 |
| spectrin beta, non-erythrocytic 1 | 20742 |
| trafficking protein, kinesin binding 1 | 67095 |
| leucine rich repeat and fibronectin type III domain containing 5 | 238205 |
| N-myc downstream regulated gene 3 | 29812 |
| ethanolamine kinase 1 | 75320 |
| post-GPI attachment to proteins 1 | 241062 |
| protein phosphatase 3, regulatory subunit B, alpha isoform (calcineurin B, type I) | 19058 |
| protein tyrosine phosphatase-like A domain containing 1 | 57874 |
| kelch repeat and BTB (POZ) domain containing 7 | 211255 |
| sema domain, transmembrane domain (TM), and cytoplasmic domain, (semaphorin) 6D | 214968 |
| TMEM9 domain family, member B | 56786 |
| hyaluronic acid binding protein 4 | 56541 |
| family with sequence similarity 13, member B | 225358 |
| EP300 interacting inhibitor of differentiation 1 | 58521 |
| small nuclear ribonucleoprotein N | 20646 |
| EH domain binding protein 1 | 216565 |
| A kinase (PRKA) anchor protein (yotiao) 9 | 100986 |
| calmodulin 3 | 12315 |
| stathmin-like 2 | 20257 |
| zinc finger, DHHC domain containing 21 | 68268 |
| iduronate 2-sulfatase | 15931 |
| solute carrier family 25 (mitochondrial carrier), member 18 | 71803 |
| potassium inwardly-rectifying channel, subfamily J, member 10 | 16513 |
| glial cell line derived neurotrophic factor family receptor alpha 2 | 14586 |
| SPARC-like 1 | 13602 |
| tetratricopeptide repeat domain 9 | 69480 |
| glycogen synthase kinase 3 beta | 56637 |
| calmodulin regulated spectrin-associated protein 1 | 227634 |
| sorbin and SH3 domain containing 1 | 20411 |
| WW domain containing adaptor with coiled-coil | 225131 |
| cystatin C | 13010 |
| heterochromatin protein 1, binding protein 3 | 15441 |
| calpain 7 | 12339 |
| tetratricopeptide repeat domain 3 | 22129 |
| Ral GTPase activating protein, alpha subunit 1 | 56784 |
| chromodomain helicase DNA binding protein 9 | 109151 |
| spastin | 50850 |
| epidermal growth factor receptor pathway substrate 15 | 13858 |
| thymocyte selection-associated high mobility group box | 252838 |
| fibroblast growth factor 14 | 14169 |
| diphosphoinositol pentakisphosphate kinase 1 | 327655 |
| diazepam binding inhibitor | 13167 |
| ATPase, H+ transporting, lysosomal V1 subunit B2 | 11966 |
| ubiquitin protein ligase E3 component n-recognin 3 | 68795 |
| RIKEN cDNA 2310061I04 gene | 69662 |
| pleiotrophin | 19242 |
| RAB GTPase activating protein 1-like | 29809 |
| tankyrase, TRF1-interacting ankyrin-related ADP-ribose polymerase | 21951 |
| patatin-like phospholipase domain containing 8 | 67452 |
| zinc finger, RAN-binding domain containing 1 | 360216 |
| ORAI calcium release-activated calcium modulator 2 | 269717 |
| transformation related protein 53 binding protein 1 | 27223 |
| prosaposin | 19156 |
| zinc finger protein 148 | 22661 |
| acyl-CoA synthetase bubblegum family member 1 | 94180 |
| failed axon connections homolog (Drosophila) | 76132 |
| DnaJ (Hsp40) homolog, subfamily B, member 14///RIKEN cDNA 1110018F16 gene | 70604///68594 |
| phosphatase and tensin homolog | 19211 |
| scrapie responsive gene 1 | 20284 |
| RAR-related orphan receptor beta | 225998 |
| selenoprotein W, muscle 1 | 20364 |
| cystathionine beta-synthase | 12411 |
| lectin, galactose binding, soluble 8 | 56048 |
| nebulette | 74103 |
| immunoglobulin superfamily, member 1 | 209268 |

**Downregulated**

| **Gen.title** | **Gene.ID** |
| --- | --- |
| protein kinase D3 | 75292 |
| nuclear distribution gene E homolog 1 (A nidulans) | 67203 |
| SEH1-like (S. cerevisiae | 72124 |
| asp (abnormal spindle)-like, microcephaly associated (Drosophila) | 12316 |
| actinin, alpha 1 | 109711 |
| OAF homolog (Drosophila) | 102644 |
| palladin, cytoskeletal associated protein | 72333 |
| far upstream element (FUSE) binding protein 1 | 51886 |
| inhibitor of DNA binding 2 | 15902 |
| Rho-related BTB domain containing 3 | 73296 |
| matrin 3 | 17184 |
| spectrin repeat containing, nuclear envelope 2 | 319565 |
| spectrin beta, non-erythrocytic 1 | 20742 |
| septin 6 | 56526 |
| RAN binding protein 2 | 19386 |
| SET nuclear oncogene | 56086 |
| isoleucine-tRNA synthetase | 105148 |
| tyrosyl-DNA phosphodiesterase 2 | 56196 |
| predicted gene, 20253///cell cycle associated protein 1 | 100504494///53872 |
| low density lipoprotein receptor | 16835 |
| GRP1 (general receptor for phosphoinositides 1)-associated scaffold protein | 56149 |
| Hbs1-like (S. cerevisiae) | 56422 |
| metadherin | 67154 |
| LSM14 homolog A (SCD6, S. cerevisiae) | 67070 |
| CCR4-NOT transcription complex, subunit 6 | 104625 |
| promyelocytic leukemia | 18854 |
| ubiquitin-like modifier activating enzyme 2 | 50995 |
| nuclear casein kinase and cyclin-dependent kinase substrate 1 | 98415 |
| eukaryotic translation initiation factor 5 | 217869 |
| TAR DNA binding protein | 230908 |
| periphilin 1 | 223828 |
| melanoma associated antigen (mutated) 1-like 1 | 245631 |
| zinc finger protein 207 | 22680 |
| squamous cell carcinoma antigen recognized by T cells 3 | 53890 |
| RIKEN cDNA C330006P03 gene///homer homolog 1 (Drosophila) | 320588///26556 |
| additional sex combs like 1 | 228790 |
| mediator complex subunit 1 | 19014 |
| cleavage and polyadenylation specific factor 6 | 432508 |
| TDP-glucose 4,6-dehydratase | 76355 |
| cytidine 5'-triphosphate synthase | 51797 |
| poly(A) binding protein, nuclear 1 | 54196 |
| adenylate kinase 4 | 11639 |
| Yy2 transcription factor///membrane-bound transcription factor peptidase, site 2 | 100073351///270669 |
| cellular nucleic acid binding protein | 12785 |
| RNA binding motif protein 12 | 75710 |
| polymerase (RNA) II (DNA directed) polypeptide B | 231329 |
| meteorin, glial cell differentiation regulator | 70083 |
| p53 induced death domain protein 1 | 57913 |
| BTAF1 RNA polymerase II, B-TFIID transcription factor-associated, (Mot1 homolog, S. cerevisiae) | 107182 |
| solute carrier family 20, member 2 | 20516 |
| fibronectin leucine rich transmembrane protein 3 | 71436 |
| transcription elongation regulator 1 (CA150) | 56070 |
| protocadherin 7 | 54216 |
| utrophin | 22288 |
| C-terminal binding protein 2 | 13017 |
| NMD3 homolog (S. cerevisiae) | 97112 |
| katanin p80 subunit B like 1 | 72425 |
| CCR4-NOT transcription complex, subunit 2 | 72068 |
| PDS5, regulator of cohesion maintenance, homolog A (S. cerevisiae) | 71521 |
| jumonji, AT rich interactive domain 2 | 16468 |
| DEAD (Asp-Glu-Ala-Asp) box polypeptide 19a | 13680 |
| allograft inflammatory factor 1-like | 108897 |
| chromodomain helicase DNA binding protein 1 | 12648 |
